# Non-destructive identification of single hard seed via multispectral imaging analysis in six legume species

**DOI:** 10.1186/s13007-020-00659-5

**Published:** 2020-08-26

**Authors:** Xiaowen Hu, Lingjie Yang, Zuxin Zhang

**Affiliations:** State Key Laboratory of Grassland Agro-ecosystems, Key Laboratory of Grassland Livestock Industry Innovation, Ministry of Agriculture and Rural Affairs, Engineering Research Center of Grassland Industry, Ministry of Education, College of Pastoral Agriculture Science and Technology, Lanzhou University, Lanzhou, 730000 China

**Keywords:** Hard seed, Legume species, Multispectral imaging, Multivariate analysis

## Abstract

**Background:**

Physical dormancy (hard seed) occurs in most species of Leguminosae family and has great consequences not only for ecological adaptation but also for agricultural practice of these species. A rapid, nondestructive and on-site screening method to detect hard seed within species is fundamental important for maintaining seed vigor and germplasm storage as well as understanding seed adaptation to various environment. In this study, the potential of multispectral imaging with object-wise multivariate image analysis was evaluated as a way to identify hard and soft seeds in *Acacia seyal*, *Galega orientulis*, *Glycyrrhiza glabra*, *Medicago sativa*, *Melilotus officinalis*, and *Thermopsis lanceolata*. Principal component analysis (PCA), linear discrimination analysis (LDA), and support vector machines (SVM) methods were applied to classify hard and soft seeds according to their morphological features and spectral traits.

**Results:**

The performance of discrimination model via multispectral imaging analysis was varied with species. For *M. officinalis*, *M. sativa*, and *G. orientulis*, an excellent classification could be achieved in an independent validation data set. LDA model had the best calibration and validation abilities with the accuracy up to 90% for *M. sativa*. SVM got excellent seed discrimination results with classification accuracy of 91.67% and 87.5% for *M. officinalis* and *G. orientulis*, respectively. However, both LDA and SVM model failed to discriminate hard and soft seeds in *A. seyal*, *G. glabra*, and *T. lanceolate*.

**Conclusions:**

Multispectral imaging together with multivariate analysis could be a promising technique to identify single hard seed in some legume species with high efficiency. More legume species with physical dormancy need to be studied in future research to extend the use of multispectral imaging techniques.

## Background

Physical dormancy (PY, referred as hard seed) occurs in at least 18 angiosperm plant families including Fabaceae [1, 2], and is caused by a water-impermeable seed or fruit coat [1, 3, 4]. This kind of dormancy prevents seeds from imbibing water even under favorable environmental conditions, and it may play a role in determining the time and place of seed germination in the field. Also, physical dormancy may help to ensure long-term seed survival, especially for wild species growing in harsh environments [5]. For example, the storage life of physical dormant soybean seeds is longer than those of nondormant seeds [6]. Furthermore, physical dormant seeds generally exhibit a greater vigor than those without physical dormancy in *Codariocalyx motorius* [7], *Glycyrrhiza uralensis* [8], and *Lespedeza bicolor* [9]. However, from agronomic perspectives, physical dormancy is an undesirable trait because it prevents rapid imbibition and synchronous germination, leading to non-uniform seedling establishment [10]. Therefore, distinguishing seeds with and without physical dormancy has great practical significance, as it is important to seed vigor and germplasm storage as well as understanding seed adaptation to various environment.

Since hard seed is impermeable to water, distinguishing whether a seed imbibed or not when soaking in the water is the most common method to determine seed physical dormancy [1, 11]. However, this process destroys the seed coat structure of soft seeds and thus not suitable for online measurements and sorting. Moreover, this method is very time consuming as it often takes several days to a month to detect presence of physical dormancy depending on species [12]. Thus, a rapid, nondestructive and on-site screening method to detect hard seed is necessary not only for research purpose but also for seed grading and sorting in seed industry.

Morphological, structural and compositional properties of seed coat have been reported to affect seed dormancy status [3, 4, 13–15]. The intraspecific or even intra-individual variation in seed size have been found to influence seed dormancy status [1, 16]. Also, seed coat compositional properties such as polyphenols content including flavonoids, lignin and lignans showed a positive relationship with dormancy in faba bean [17] and pea [14]. These results imply discriminating soft and hard seeds through their morphological and compositional traits is possible. Indeed, previous studies [18] found that near infrared spectroscopy can provide a high accuracy in identifying hard seeds of three legume species. However, this method did not apply seed image analysis techniques, and seed spectral traits was measured individually which was time consuming and impractical.

Multispectral imaging is an emerging technology that integrates conventional imaging and spectroscopy to simultaneously attain both spatial and spectral information of an object [19]. The merits of nondestructive, straightforward measurement strategies that do not require pre-treatment make multispectral imaging analysis ideally suited for online process monitoring and quality control. Recently, this technique has been increasingly used to assess food safety and quality, such as contaminant detection, defect identification, constituent analysis, and quality evaluation [20, 21]. In regard of seed identification, multispectral imaging was originally applied to discrimination of transgenic rice seeds from its non-transgenic counterparts [20], discrimination of rice seeds among different varieties [22], and classification of maize kernels [23]. Refer to the potential morphological and chemical difference among hard seed and soft seed, multispectral imaging may have a great potential in distinguishing seeds with or without physical dormancy.

Six common legume species including *Acacia seyal*, *Galega orientulis*, *Glycyrrhiza glabra*, *Medicago sativa*, *Melilotus officinalis*, and *Thermopsis lanceolata* were applied in this study. Among these species, *G. orientulis*, *M. sativa*, and *M. officinalis* [24, 25] are important forage species, which are widely cultivated in the world. *G. glabra* [26] and *T. lanceolate* [27] have been used as traditional Chinese medicine. *A. seyal* has medical and ecological value [28]. According to previous studies [24–28], seeds of these six species exhibited physical dormancy which restrict their cultivation. Thus, discriminating hard and soft seeds for these species is extremely important not only for research purpose but also for practical significance.

Herein, we described a new approach with merits of nondestructive, rapid and high throughput to discriminate hard and soft seeds of legume species, based on the VideometerLab 4 spectral imaging system in combination with multivariate analysis.

## Results

### Morphologic feathers of hard and soft seeds

The difference in morphological traits between hard and soft seeds of a species was varied with species (Table 1). For *M. sativa* and *M. officinalis*, a significant difference was observed between hard and soft seeds of each species in terms of the mean value of seed area, length, Width/Length Ratio, compactness circle, BetaShape a, BetaShape b, CIELab L*, CIELab a*, CIELab b*, and saturation, while no significant difference existed in terms of the mean value of compactness ellipse and vertical orientation. However, for *A.seyal*, *G. glabra* and *T. lanceolate*, almost all morphological traits except for area of *T. lanceolate* and Width/Length Ratio, and CIELab L* of *G. glabra*, showed no significant difference between hard seed and soft seed within each species. For *G. orientulis*, a significant difference was found between hard seed and soft seed in terms of the mean value of seed area, length, compactness ellipse, CIELab a* and hue, while no significant difference existed in terms of length, width, Width/Length Ratio, compactness circle, BetaShape a, BetaShape b, vertical skewness, CIELab L*, CIELab b*, and saturation.

**Table 1:** Morphological features of hard and soft seeds for six species

	*Acacia seyal*		*Galega orientulis*		*Glycyrrhiza glabra*		*Medicago sativa*		*Melilotus officinalis*		*Thermopsis lanceolata*	
	Hard	Soft	Hard	Soft	Hard	Soft	Hard	Soft	Hard	Soft	Hard	Soft
Area (mm2)	18.06 ± 2.65	18.53 ± 2.94	5.6 ± 0.68	5.78 ± 0.67**	6.46 ± 0.76	6.55 ± 0.72	2.50 ± 0.37	2.60 ± 0.29**	2.40 ± 0.28	2.52 ± 0.28**	11.98 ± 1.43	11.65 ± 1.28*
Length (mm)	6.12 ± 0.55	6.18 ± 0.56	3.63 ± 0.29	3.69 ± 0.27*	3.07 ± 0.20	3.1 ± 0.19	2.32 ± 0.21	2.40 ± 0.16**	2.19 ± 0.17	2.22 ± 0.17	4.31 ± 0.27	4.25 ± 0.28
Width (mm)	3.86 ± 0.34	3.92 ± 0.34	2.00 ± 0.15	2.02 ± 0.15	2.74 ± 0.16	2.74 ± 0.15	1.46 ± 0.14	1.45 ± 0.11	1.47 ± 0.09	1.52 ± 0.09**	3.62 ± 0.21	3.59 ± 0.19
Width/length ratio	0.63 ± 0.07	0.64 ± 0.07	0.55 ± 0.05	0.55 ± 0.05	0.89 ± 0.03	0.88 ± 0.04**	0.63 ± 0.07	0.61 ± 0.06**	0.67 ± 0.05	0.69 ± 0.05*	0.84 ± 0.03	0.85 ± 0.04
Compactness circle	0.63 ± 0.07	0.63 ± 0.07	0.52 ± 0.05	0.52 ± 0.04	0.88 ± 0.04	0.87 ± 0.04	0.60 ± 0.07	0.58 ± 0.06**	0.66 ± 0.05	0.67 ± 0.05*	0.82 ± 0.04	0.82 ± 0.04
Compactness ellipse	1.00 ± 0.00	1.00 ± 0.00	0.99 ± 0.01	0.99 ± 0.01*	1.00 ± 0.01	1.00 ± 0.00	0.99 ± 0.01	0.99 ± 0.01	1.00 ± 0.00	1.00 ± 0.00	0.99 ± 0.00	0.99 ± 0.00
Beta shape a	1.58 ± 0.08	1.59 ± 0.10	1.32 ± 0.07	1.32 ± 0.07	1.49 ± 0.06	1.48 ± 0.05	1.51 ± 0.15	1.46 ± 0.13**	1.62 ± 0.05	1.61 ± 0.06*	1.46 ± 0.06	1.46 ± 0.06
Beta shape b	1.48 ± 0.06	1.48 ± 0.06	1.27 ± 0.07	1.27 ± 0.06	1.42 ± 0.06	1.41 ± 0.05	1.43 ± 0.12	1.40 ± 0.12*	1.55 ± 0.06	1.54 ± 0.05**	1.36 ± 0.06	1.36 ± 0.05
Vertical Skewness	-0.05 ± 0.02	-0.06 ± 0.03	-0.03 ± 0.03	-0.03 ± 0.02	-0.04 ± 0.02	-0.04 ± 0.02	-0.04 ± 0.04	-0.03 ± 0.03**	-0.03 ± 0.02	-0.04 ± 0.02	-0.05 ± 0.02	-0.06 ± 0.02
CIELab L*	35.12 ± 2.84	35.07 ± 2.37	41.30 ± 3.07	40.9 ± 3.44	36.70 ± 3.27	37.66 ± 3.33*	47.89 ± 3.62	51.83 ± 2.72**	45.16 ± 3.96	48.92 ± 2.66**	26.69 ± 2.04	27.01 ± 1.89
CIELab a*	6.94 ± 2.30	6.19 ± 2.06*	11.75 ± 1.33	12.86 ± 1.45**	4.77 ± 1.92	4.39 ± 1.80	10.05 ± 1.80	8.96 ± 1.61**	10.98 ± 2.01	10.44 ± 1.90*	-0.19 ± 0.58	-0.18 ± 0.52
CIELab b*	14.43 ± 1.91	14.11 ± 1.40	27.30 ± 3.15	26.73 ± 3.44	15.08 ± 2.37	15.05 ± 1.89	24.64 ± 4.12	20.52 ± 2.40**	27.46 ± 3.58	26.35 ± 2.63**	3.38 ± 1.21	3.14 ± 0.82*
Saturation	16.11 ± 2.46	15.52 ± 1.69	29.78 ± 2.86	29.74 ± 2.99	15.89 ± 2.62	15.75 ± 2.08	26.68 ± 4.07	22.48 ± 2.05**	29.63 ± 3.69	28.40 ± 2.64**	3.44 ± 1.22	3.19 ± 0.81*
Hue	1.13 ± 0.10	1.16 ± 0.12	1.16 ± 0.06	1.12 ± 0.08**	1.27 ± 0.10	1.29 ± 0.10*	1.18 ± 0.08	1.16 ± 0.09**	1.19 ± 0.06	1.19 ± 0.07**	-0.6 ± 1.31	-0.4 ± 1.38
Vertical orientation	0.01 ± 0.09	-0.01 ± 0.07	0.00 ± 0.11	0.00 ± 0.12	0.00 ± 0.11	-0.01 ± 0.11	0.01 ± 0.1	0.00 ± 0.09	0.00 ± 0.13	0.00 ± 0.11	0.02 ± 0.08	0.01 ± 0.09

* and ** indicate significant difference at 0.05 and 0.01 levels, respectively, between hard and soft seeds for each species based on the Independent T test

Table 1 Morphological features of hard and soft seeds for six species.

### Spectroscopic analysis of hard and soft seeds

**Fig. 1:**
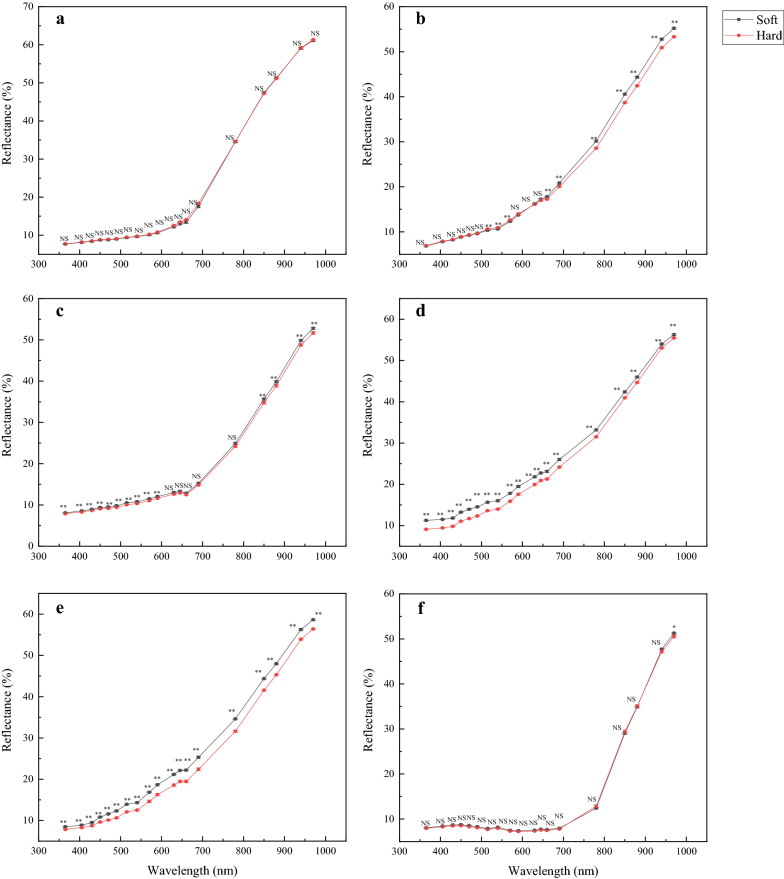
Mean light reflectance of 19 wavelengths (nm) in hard and soft seeds. **a** *Acacia seyal*, **b** *Galega orientulis*, **c** *Glycyrrhiza glabra*, **d** *Medicago sativa*, **e** *Melilotus officinalis*, and **f** *Thermopsis lanceolata*. * and ** indicate significant difference between hard and soft seeds at 0.05 and 0.01 levels, respectively. NS indicate no significant difference between two species. Vertical bars represent standard deviations

Except for *A.seyal*, the spectroscopic analysis revealed a significant difference between hard and soft seeds of the other five species in the mean reflectance (Fig. 1). For *M. sativa* and *M. officinalis*, soft seeds have significant higher reflectance than those of hard seeds in the whole wavelength region. Consistent with *M. sativa* and *M. officinalis*, soft seeds of *G. glabra* also showed a higher reflectance than hard seeds, while the statistical significance was observed only in the spectral range from 405 nm to 590 nm and from 850 nm to 970 nm. Contrast with above, the soft seeds of *G. orientulis* showed a significant lower reflectance than the hard seeds in the spectral range from 515 nm to 570 nm, while an opposite trend was observed in the spectral range from 660 nm to 970 nm. For *T. lanceolate*, no significant difference was detected in the reflectance in the whole spectral range except for in 970 nm.

### Principal component analysis (PCA)

There was no distinct difference in terms of PCA score between hard and soft seeds for all species regardless of dimensionality applied (Additional file 1: Figure S1, Additional file 2: Figure S2 and Additional file 3: Figure S3). Here, we took the first two principal components as an example.

**Fig. 2:**
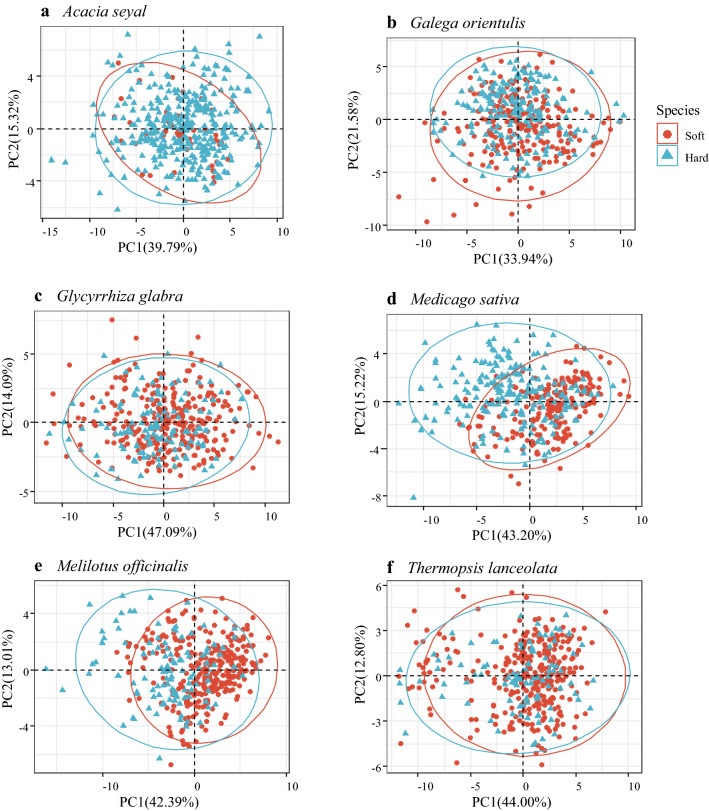
Two-dimensional biplot of the first two PCs for morphological and spectral features data set in hard and soft seeds. Note: Blue color represents for hard and red for soft seed. **a** *Acacia seyal*, **b** *Galega orientulis*, **c** *Glycyrrhiza glabra*, **d** *Medicago sativa*, **e** *Melilotus officinalis* and **f** *Thermopsis lanceolata*

The first two principle components extracted from the morphological and spectral traits explained 55.11%, 55.52%, 61.18%, 57.42%, 55.40% and 56.80% of the original variance for *A. seyal*, *G. orientulis*, *G. glabra*, *M. officinalis*, *M. sativa*, and *T. lanceolate*, respectively (Fig. 2). However, the biplot of PCA for either of the species did not reveal a distinct separation between hard and soft seeds, suggesting that discrimination between these two kinds of seed within species through PCA is difficult.

### Seed classification based on linear discrimination analysis (LDA) model

The performance of LDA model in classifying hard seeds and soft seeds was varied with species (Table 2). For *M. officinalis*, *M. sativa*, and *G. orientulis*, LDA model had a high average accuracy value of 90%, 90% and 85%, respectively in classifying hard and soft seeds in independent validation data sets. Meanwhile, the sensitivity and specificity for hard seed classification in these three species were reasonably good with a range from 82.69% to 86.67%, and from 84.29% to 95.59%, respectively, for independent validation data sets. For *G. glabra* and *T. lanceolate*, a high classification accuracy and specificity was observed in both species for independent validation data sets, while the classification specificity for hard seeds was quite low with value of 50% and 33.33%, respectively. Contrast with this, the average correct classification and specificity for *A. seyal* was 87.5% and 98.11%, respectively. However, the classification specificity for hard seeds of *A. seyal* was only 7.14%.

**Table 2:** Performance of linear discrimination analysis (LDA) model in identifying single hard seed in six species

Species	Accuracy (%)	Sensitivity (%)	Specificity (%)
*Acacia seyal*			
Calibration	91.79	99.60	26.67
CV	86.79	96.80	3.33
Validation	87.50	98.11	7.14
*Galega orientulis*			
Calibration	91.07	88.72	93.20
CV	86.07	84.21	87.76
Validation	85.00	86.00	84.29
*Glycyrrhiza glabra*			
Calibration	82.86	60.00	92.82
CV	72.14	40.00	86.15
Validation	77.50	50.00	87.50
*Medicago sativa*			
Calibration	92.86	89.58	96.32
CV	88.93	86.81	91.18
Validation	90.00	82.69	95.59
*Melilotus officinalis*			
Calibration	95.36	89.74	97.52
CV	92.14	83.33	95.54
Validation	90.00	86.67	91.11
*Thermopsis lanceolata*			
Calibration	86.07	55.38	95.35
CV	80.00	38.46	92.56
Validation	75.00	33.33	87.10

*CV* cross validation

**Fig. 3:**
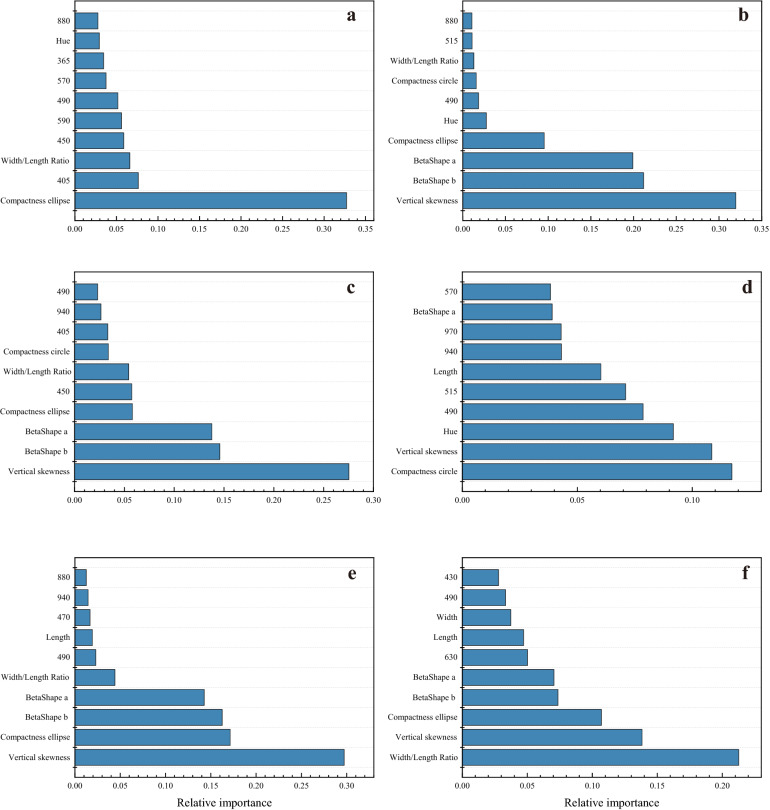
Relative importance of morphological and spectral features for linear discrimination analysis model. **a** *Acacia seyal*, **b** *Galega orientulis*, **c** *Glycyrrhiza glabra*, **d** *Medicago sativa*, **e** *Melilotus officinalis* and **f** *Thermopsis lanceolata*

There was a large variation for the contribution of each trait on LDA model across species (Fig. 3). For *M. sativa*, the compactness circle explained 11.7% of the total variation, followed by vertical skewness (10.8%), hue (9.2%), 490 nm (7.9%) and 515 nm (7.1%). For *M. officinalis*, the vertical skewness explained 29.7% of the total variation, followed by compactness ellipse (17.1%), BetaShape b (16.3%), BetaShape a (14.3%) and Width/Length Ratio, (4.4%) (Fig. 3). Similarly, for *M. officinalis*, the vertical skewness explained 32.0% of the total variation of *G. orientulis*, followed by BetaShape b (21.2%), BetaShape a (19.9%), compactness ellipse (9.5%) and hue (2.8%) (Fig. 3).

### Seed classification based on support vector machine (SVM) model

In agreement with the LDA model, the performance of SVM model in classifying hard and soft seeds differed among species (Table 3). SVM model had an average accuracy value as high as 91.67%, 89.17% and 87.5% in seed classification for independent validation data sets of *M. officinalis*, *M. sativa*, and *G. orientulis*, respectively. Meanwhile, the sensitivity and specificity for hard seed classification in these three species were reasonably good with a range from 76.67% to 88%, and from 87.14% to 96.67%, respectively. For *A. seyal*, *G. glabra*, and *T. lanceolate*, the average classification accuracy was 88.33%, 80% and 77.5%, respectively. However, the classification sensitivity for hard seeds in *G. glabra*, and *T. lanceolate* was quite low with value of 46.88% and 7.41%, respectively. Similarly, for classification sensitivity, the classification specificity for *A. seyal* was 0.

**Table 3:** Performance of support vector machine (SVM) model in identifying single hard seed in six species

Species	Accuracy (%)	Sensitivity (%)	Specificity (%)
*Acacia seyal*			
Calibration	89.29	100.00	0.00
CV	89.29	100.00	0.00
Validation	88.33	100.00	0.00
*Galega orientulis*			
Calibration	91.79	90.98	92.52
CV	89.29	87.22	91.16
Validation	87.50	88.00	87.14
*Glycyrrhiza glabra*			
Calibration	82.14	57.65	92.82
CV	67.50	4.71	94.87
Validation	80.00	46.88	92.05
*Medicago sativa*			
Calibration	95.00	93.75	96.32
CV	86.79	83.33	90.44
Validation	89.17	84.62	92.65
*Melilotus officinalis*			
Calibration	90.36	73.08	97.03
CV	88.57	71.79	95.05
Validation	91.67	76.67	96.67
*Thermopsis lanceolata*			
Calibration	80.71	20.00	99.07
CV	80.00	20.00	98.14
Validation	77.50	7.41	97.85

*CV*  cross validation

**Fig. 4:**
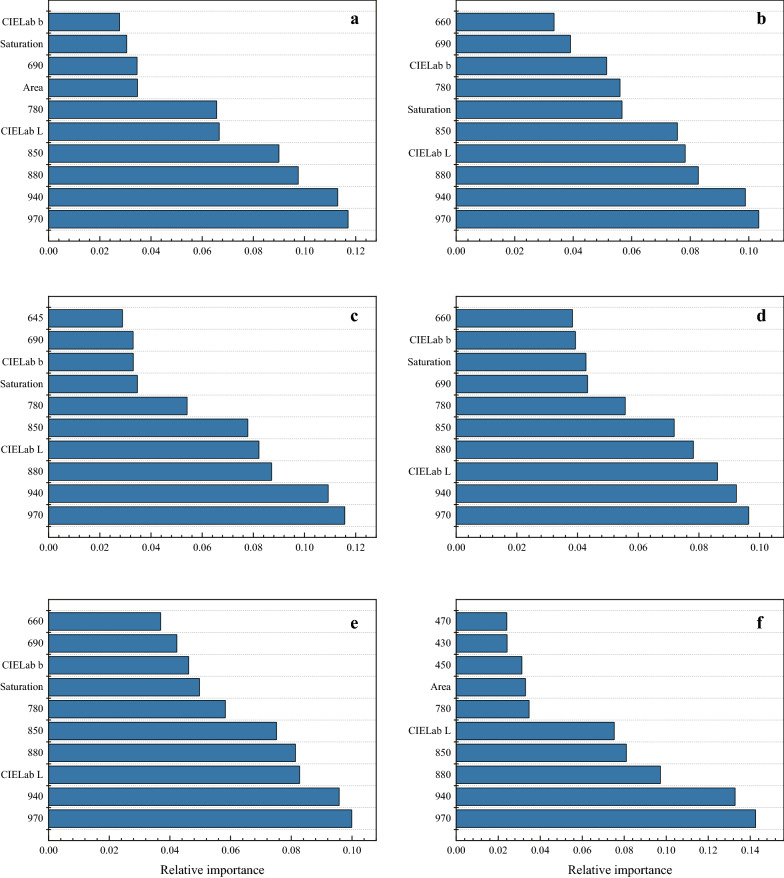
Relative importance of morphological and spectral features for support vector machines model. **a** *Acacia seyal*, **b** *Galega orientulis*, **c** *Glycyrrhiza glabra*, **d** *Medicago sativa*, **e** *Melilotus officinalis*, and **f** *Thermopsis lanceolata*

For all species, the reflectance in the near infrared region (840–970 nm) contributed more than morphological traits for SVM model. For example, the reflectance in 970 nm, 940 nm, 880 nm and 850 nm ranked the first five traits contribute to SVM model, and explained 35.2%, 33.9% and 36.1% of the total variation for *M. officinalis*, *M. sativa* and *G. orientulis*, respectively (Fig. 4).

## Discussion

Previous studies [1, 14, 17] have indicated that morphological and spectral traits of a species may differ between hard and soft seeds, and thus can be employed as a tool for seed classification. Consistent with this, our study clearly shows that there is a significant difference in at least one of morphological and spectral traits between hard and soft in six tested species. However, it is worth noting that an overlap exists between hard seed and soft seeds, though significant difference is observed in terms of the mean value, suggesting that it is not appropriate to discriminate hard and soft seed of a species with any single trait. Moreover, the difference between hard and soft seed is varied a lot across different species. For example, hard and soft seeds of *M. officinalis*, *M. sativa* and *G. orientulis* have significant difference in most traits measured both in morphological and spectral. However, for the other three species, significant difference between hard and soft seeds is only detected in a very few traits.

Also, no consistent difference is observed between hard and soft seeds among species. For example, hard seeds of *M. officinalis*, *M. sativa* and *G. orientulis* are smaller than soft seeds, while the opposite trend is observed in *T. lanceolate*. Besides, soft seeds of *M. officinalis*, *M. sativa* and *G. glabra* have a higher reflectance in the whole wavelength region than hard seeds, and an opposite trend is observed in the short wavelength region (365–590 nm) in *G. orientulis*. These variations among species may also explain the performance difference of discrimination model on different species.

It is clearly to see that from the PCA scatter plot the PCA method could not separate hard and soft seeds in all test species. A possible reason is that PCA method aims to maximize the variance of variables rather than to maximize the discriminability of hard and soft seeds. In this case, if the variables between groups have very similar mean value with a large variation, the total variance will be mainly composed of variance within groups but not those between groups. Thus, PCA would not detect the difference among groups. Indeed, either for morphological or spectral trait, they all have very close mean value with a large overlap distribution between hard and soft seeds in six species. Also, we notice that a part of information has been lost after PCA analysis, since the first two principle components only explained the total variance ranged from 55.11% to 62.69%, the loss of information may further lead to the failure to separate groups by PCA.

Unlike PCA, the supervised methods such as LDA and SVM, aim to minimize the distance within classes and to maximize the distance between groups, thus they showed good discriminability among groups [29–31]. Consistent with this, our study shows that both LDA and SVM model provide a high classification accuracy for hard and soft seeds in *M. officinalis*, *M. sativa* and *G. orientulis*. It is interesting that, although both the LDA and SVM models have a high accuracy in seed discrimination, they seem to work in completely different ways. When we take a close look at the relative importance of each feature, the SVM model mainly relies on NIR region spectral trait in model building since spectral traits contribute more in the model, while LDA focus more on seed morphology which contribute most in LDA discrimination model building. Hu et al. [32] also had a remarkably similar finding in seed discrimination between alfalfa and sweet clover via multispectral imaging analysis. However, we failed to detect the reason in depth for this differentiation between these two methods. Further study involving methods combining LDA and SVM may get higher accuracy results for multispectral analysis of hard seeds.

Contrast with this, although the classification accuracy is reasonably good in *A. seyal*, *G. glabra*, and *T. lanceolate*, the model is less specificity or sensitive since the classification specificity for hard seeds in *A. seyal* is only 7.14%, and the sensitivity for hard seeds in *G. glabra*, and *T. lanceolate* is only 50% and 33.33%, respectively. In the former case, most soft seeds are misclassified as hard seeds; while in the latter case, the model will classify hard seeds as soft seeds in a high probability. This inconsistence between classification accuracy and sensitivity or specificity are mainly attributed to the unbalanced data set. For instance, the number of hard and soft seeds of *A. seyal* were unbalanced with 30 and 250 in the calibration set, and 14 and 106 in the independent validation data set. In this case, when the model classified most seeds as hard seed, the model will still have a high average classification accuracy and sensitivity, but with a very low specificity. However, it is worth noting that the unbalanced data is not the reason for poor performance of the model since there is no reliable empirical evidence to support the claim that unbalanced data set has a negative effect on the performance of LDA [29]. Indeed, when some hard seeds of *A. seyal* were randomly removed from the sample, the average classification accuracy and sensitivity is decreased and consequently increases the classification specificity. These results suggest current model used in our study could not discriminate hard and soft seeds in *A. seyal*, *G. glabra*, and *T. lanceolate*. This is possibly due to the difference in morphological and spectral traits between hard and soft seeds is not big enough in our study. Sun et al. [33] reported that using near infrared spectroscopy can provide a high accuracy in identifying hard seeds in *G. uralensis*, and their results showed a significant difference in light absorbance when the wavelength is higher than 1000 nm. Consistent with this, our study also showed that the difference in spectral trait between hard and soft seeds in *G. glabra* increased as the wavelength increasing. In addition, a significant difference between hard and soft seeds in *A. seyal* is detected only at 970 nm. Thus, a wide range wavelength such as near infrared spectroscopy may help to improve the data quality and favor discrimination model building. Furthermore, other machine learning tools, such as random forest (RF) and back propagation neural network (BPNN), which have been proved to be effective in discrimination of soybean seeds [22] and high-quality watermelon seeds [21], can be applied in separating hard and soft seeds in future studies.

## Materials and methods

### Seed sample

**Fig. 5:**
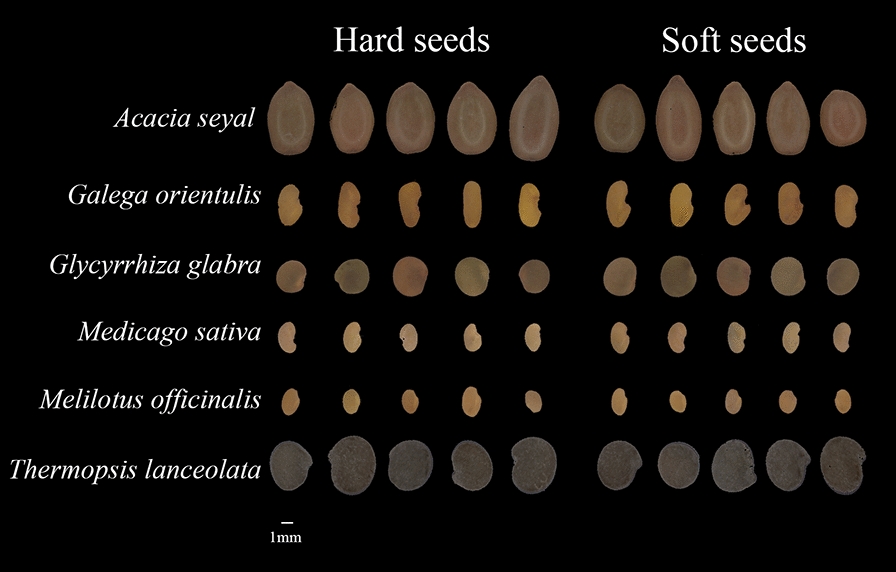
Images of hard and soft seeds of six species

Seeds of *Acacia seyal*, *Galega orientulis*, *Glycyrrhiza glabra*, *Medicago sativa*, *Melilotus officinalis*, and *Thermopsis lanceolata* (Fig. 5) were provided by the Official Herbage and Turfgrass Seeds Testing Center, Ministry of Agriculture and Rural Affairs, China. Seeds were kept in water-proof bags in laboratory conditions (20 °C, 35% relative humidity) till the time of image acquisition in April 2019. The initial moisture content of *A. seyal*, *G. orientulis*, *G. glabra*, *M. sativa*, *M. officinalis*, and *T. lanceolate* were 8.5%, 7.3%, 6.8%, 6.5%, 6.7% and 8.1%, respectively.

The amount of seeds used for the experiment of hard and soft seed classification was 400. For each species, 280 seeds for each sample were randomly selected as calibration set and the remaining 120 seeds were used for independent validation set.

### Multispectral imaging system

Multispectral images were acquired with a VideometerLab4 (Videometer, Hørsholm, Denmark) multispectral imaging system. The samples of 400 seeds for each species in each petri dish were placed beneath a hollow integrating sphere, with a camera located in the top of the sphere. During image capture, the sphere closes over the sample stage to create optically closed conditions, allowing even lighting with minimal shadows and specular reflection. Samples were illuminated by 19 high power light emitting diodes (LEDs) at specific wavelengths: 365, 405, 430, 450, 470, 490, 515, 540, 570, 590, 630, 645, 660, 690, 780, 850, 880, 890, and 970 nm. The LEDs strobe successively in a scan time of approximately five seconds, resulting in a monochrome image at each wavelength at 19 different wavelengths. The images consisted of 2192 × 2192 pixels, with a high spatial resolution of approximately 40 μm/pixel. Before acquiring multispectral images, the system was fully calibrated radiometrically and geometrically by using three successive plates: a white one for reflectance correction, a dark one for background correction and a doted one for geometric pixel position aligning calibration, followed by a light setup calibration.

### Determination of hard seed

Following imaging acquisition, each seed was placed on two sheets of filter paper (Hangzhou Shuangquan, Hangzhou, Zhejiang, China) moistened with 10 ml distilled water in 12-cm-diameter petri dishes and incubated at 20 °C for 14 days. The number of imbibed (soft seed) and unimbibed (hard seed) seeds in each dish was monitored daily. When a seed imbibed, there was a visible change in its size/volume, thus imbibed and unimbibed seeds could easily be distinguished from each other. The number of true hard and soft seeds for each data set was shown in Table 4.

**Table 4:** Number of hard and soft seeds for each data set of six species

	*Acacia seyal*	*Galega orientulis*	*Glycyrrhiza glabra*	*Medicago sativa*	*Melilotus officinalis*	*Thermopsis lanceolata*
Calibration (*n* = 280)						
Hard	250	133	85	144	78	65
Soft	30	147	195	136	202	215
Validation (*n* = 120)						
Hard	106	50	32	52	30	27
Soft	14	70	88	68	90	93

### Multispectral image analysis

The main objects appeared in the acquired multispectral image are the seeds in addition to some other objects, such as the Petri dish and its surrounding background that should be removed from the image before extracting spectral information of the individual seeds. Image segmentation was performed using the VideometerLab software version 3.10. To remove the image background, all items, except the seeds, were removed by a normalized canonical discriminant analysis (nCDA) [34] and segmented using a simple threshold. Then, the attributes of the seeds such as morphological traits and main spectral features of all individual seeds were extracted from the image analysis and processing. The morphological traits included area, length, width, Width/Length Ratio, compactness circle, compactness ellipse, BetaShape a, BetaShape b, vertical skewness, CIELab L*, CIELab a*, CIELab b*, saturation, hue and vertical orientation [19, 35]. Explanation of morphological traits were listed in Additional file 4: Table S1. The extracted spectral signatures of the seeds represent the mean intensity of the reflected light at each single wavelength calculated from all seed pixels in the image.

### Multivariate data analysis

Multivariate analysis including PCA, LDA and support vector machines (SVM) using *FactoMineR, MASS, and e1071 package in R* respectively in the present study to classify and screen the seeds.

#### PCA

To identify the patterns hidden in the extracted morphological features and spectral data of all seeds, PCA was carried out as an explorative multivariate data analysis technique, which commonly used to get an overview of the systematic variation in the data and to explore the possibility of grouping the seeds of similar morphology and spectral profiles [36–38]. PCA score was calculated based on the first two, three and all PCs.

#### LDA

LDA is a well-known algorithm, which calculates a surface separating the sample groups, by establishing a linear discriminant function that maximizes the ratio of the between class and the within-class variances [37]. In this study, the seeds were randomly sampled as calibration (70% of total sample) and validation sets (remained 30%) as shown in Table 4, LDA classification models were developed using the calibration set, and the models obtained were validated using the independent validation set, which was not used during model building. To reduce the potential overfitting, the LDA models were developed under cross-validation using leave-one-out cross-validation method in which one seed was taken out at a time, and the LDA model was built for the remaining seeds. The model was then used to classify the seed left out, and the same routine was repeated until all seeds were removed [39].

The classification method performance was evaluated by the ability to detect the presence of hard seeds in seed lots of each species through the sensitivity (eq. 1), specificity (eq. 2), and accuracy (eq. 3).

1 $$Sensitivity\,\left( \% \right)\, = \,100\,\, \times \,TH\text{ / }\left( {TH\, + \,FS} \right)$$

2 $$Specificity\left( \% \right)\, = \,100\,\, \times \,TS\text{ / }\left( {TS\, + \,FH} \right)$$

3 $$Accuracy\,\left( \% \right)\, = \,100\,\, \times \,\left( {TH\, + \,TS} \right)\text{ / }{\text{Total}}$$

where, *TH* true hard seed, *FS* false soft seed, *TS* true soft seed and *FH* false hard seed.

#### SVM

Least squares-support vector machine (SVM) is a supervised learning algorithm used for classification and regression tasks proposed by Cortes and Vapnik [40]. Compared with other analysis methods, SVM can learn in high-dimensional characteristic space with fewer calibration variables or samples, and details of the SVM algorithm can be found in previous reported research [41, 42]. It has been effectively used to perform multivariate function estimation or non-linear classification. In this study, the linear kernel was used for classification. To reduce the potential overfitting, the LS-SVM models were developed under cross-validation using leave-one-out cross-validation method as described above. The quality of classification was evaluated by calculation of sensitivity, specificity and classification accuracy as described above.

## Conclusion

In brief, our study clearly shows that multispectral imaging together with multivariate analysis could be a promising technique to identify hard seeds in some legume species with high efficiency. For *M. sativa*, LDA model has the best calibration and validation abilities with accuracy up to 90%. For *M. officinalis* and *G. orientulis*, SVM give excellent seed discrimination results with classification accuracy of 91.67% and 87.5%, respectively. However, both LDA and SVM model failed to discriminate hard and soft seeds in *A. seyal*, *G. glabra*, and *T. lanceolate*.

## Supplementary information

**Additional file 1: Figure S1.** First two principal components score for hard and soft seeds.

**Additional file 2: Figure S2.** First three principal components score for hard and soft seeds.

**Additional file 3: Figure S3.** First ten principal components score for hard and soft seeds.

**Additional file 4: Table S1.** List of the 15 extracted variables from multi spectral images.

## Data Availability

Not applicable.

## Abbreviations

CV: Cross validation
BPNN: Back propagation neural network
LDA: Linear discrimination analysis
LS-SVM: Least squares-support vector machine
PCA: Principal component analysis
RF: Random forest
SVM: Support vector machines
FS: False soft seed
FH: False hard seed
TH: True hard seed
TS: True soft seed

## References

[1] Baskin CC, Baskin JM (2014). Seeds: ecology, biogeography, and evolution of dormancy and germination.

[2] Willis CG, Baskin CC, Baskin JM, Auld JR, Venable DL, Cavender-Bares J, Donohue K, Rubio de Casas R, NESCent Germination Working Grouping. The evolution of seed dormancy: environmental cues, evolutionary hubs, and diversification of the seed plants. New Phytologist. 2014; 203:300-9. 10.1111/nph.12782 24684268

[3] Smýkal P, Vernoud V, Blair MW, Soukup A, Thompson RD (2014). The role of the testa during development and in establishment of dormancy of the legume seed. Front Plant Sci.

[4] Janská A, Pecková E, Sczepaniak B, Smýkal P, Soukup A (2018). The role of the testa during the establishment of physical dormancy in the pea seed. Ann Bot.

[5] Tran VN, Cavanagh AK. Germination and Reserve Mobilization, Chapter 1-Structural Aspects of Dormancy. 1984:1-44.

[6] Wang JL (1999). Study on preservation of soybean germplasm using soybean hard seed. Soybean Sci.

[7] Xu BM, Sun YT, Li RL, Guo C, Song YH (2006). Detection of higher vigour of hard seed of *Codariocalyx motorius*. Scientia Silvae Sinicae.

[8] Tian J, Sun Q, Wang JH, Sun BQ (2007). Differences of seed vigor among different levels of hard seeds of *Glycyrrhiza uralensis* fisch. Plant Physiol Commun.

[9] Bai CX, Han JG, Sun Y, Qin GJ (2006). Study on the relationship between hard-seededness and seed vigor of *Indigofera amblyantha* and *Lespedeza bicolor*. Acta Prataculturae Sinica.

[10] Ma F, Cholewa E, Mohamed T, Peterson CA, Gijzen M (2004). Cracks in the palisade cuticle of soybean seed coats correlate with their permeability to water. Ann Bot.

[11] Yang QH, Yin XJ, Ye WH (2006). Dormancy mechanism and breaking methods for hard seeds. Chinese Bull Botany.

[12] Hu XW, Wang YR, Wu YP (2009). Effects of the pericarp on imbibition, seed germination, and seedling establishment in seeds of *Hedysarum scoparium* Fisch et Mey. Ecolo Res.

[13] Venable DL, Brown JS (1988). The selective interactions of dispersal, dormancy, and seed size as adaptations for reducing risk in variable environments. Am Nat.

[14] Hradilová I, Trněný O, Válková M, Cechová M, Janská A, Prokešová L, Aamir K, Krezdorn N, Rotter B, Winter P, Varshney RK, Soukup A, Bednář P, Hanáček P, Smýkal P (2017). A combined comparative transcriptomic, metabolomic, and anatomical analyses of two key domestication traits: Pod dehiscence and seed dormancy in pea (*Pisum* sp.). FrontPlant Sci.

[15] Rees M (1996). Evolutionary ecology of seed dormancy and seed size. Philosophical Transact Biol Sci.

[16] Mandák B (1997). Seed heteromorphism and the life cycle of plants: a literature review. Preslia.

[17] Kantar F, Pilbeam CJ, Hebblethwaite PD (1996). Effect of tannin content of faba bean (*Vicia faba*) seed on seed vigour, germination and field emergence. Annals Appl Biol.

[18] Zhu LW, Huang YY, Wang Q, Ma HX, Sun BQ, Sun Q (2012). Nondestructive identification of hard seeds of three legume plants using near infrared spectroscopy. Transact Chinese Soc Agric Eng.

[19] Boelt B, Shrestha S, Salimi Z, Jørgensen JR, Nicolaisen M, Carstensen JM (2018). Multispectral imaging-A new tool in seed quality assessment?. Seed Sci Res.

[20] Liu C, Liu W, Lu X, Chen W, Yang J, Zheng L (2014). Nondestructive determination of transgenic *Bacillus thuringiensis* rice seeds (*Oryza sativa* L.) using multispectral imaging and chemometric methods. Food Chem.

[21] Liu W, Xu X, Liu C, Zheng L (2018). Rapid discrimination of high-quality watermelon seeds by multispectral imaging combined with chemometric methods. J Appl Spectrosc.

[22] Liu C, Liu W, Lu X, Chen W, Chen F, Yang J, Zheng L (2016). Non-destructive discrimination of conventional and glyphosate-resistant soybean seeds and their hybrid descendants using multispectral imaging and chemometric methods. J Agric Sci.

[23] Sendin K, Manley M, Williams PJ (2018). Classification of white maize defects with multispectral imaging. Food Chem.

[24] Zhang AH, Yu YW (2006). The research and utilization of *Galega orientalis* in Russia. Pratacultural Sci.

[25] Li WY, Zhang SQ, Ding SY, Shan L (2010). Root morphological variation and water use in alfalfa under drought stress. Acta Ecol Sin.

[26] Lin LC, Ruan JJ (2012). HPLC determinated the content of Glabridin from *Glycyrrhiza glabra* L. Modern Chinese Med.

[27] Yan ZZ, Li DL, Man DQ, Jiang SX, Wu H (2017). Effects of simulated drought stress by PEG6000 on seed germination of *Halimodendron halodendron* and *Thermopsis lanceolate*. Arid zone Res.

[28] Flindt C, Al-Assaf S, Phillips GO, Williams PA (2005). Studies on acacia exudate gums Part V Structural features of Acacia seyal. Food Hydrocolloids.

[29] Xue JH, Titterington DM (2008). Do unbalanced data have a negative effect on LDA?. Pattern Recogn.

[30] Ang JC, Mirzal A, Haron H, Hamed HNA (2016). Supervised, unsupervised, and semi-supervised feature selection: a review on gene selection. Transact Comput Biol Bioinform.

[31] Morra JH, Tu Z, Apostolova LG, Green AE, Toga AW, Thompson PM (2010). Comparison of adaBoost and support vector machines for detecting Alzheimer’s disease through automated hippocampal segmentation. Transact Med Imaging.

[32] Hu XW, Yang LJ, Zhang ZX, Wang YR (2020). Differentiation of alfalfa and sweet clover seeds via multispectral imaging. Seed Sci Technol.

[33] Sun Q, Li JH, Wang JH, Sun BQ (2009). Identification of hardness of licorice single seed using near infrared spectroscopy. Spectroscopy Spectral Anal.

[34] Cruz-Castillo JG, Ganeshanandam S, MacKay BR, Lawes GS, Lawoko CRO, Woolley DJ (1994). Applications of canonical discriminant analysis in horticultural research. Hortscience.

[35] Salimi Z, Boelt B. Classification of processing damage in Sugar Beet (*Beta vulgaris*). Seeds by Multispectral Image Analysis. Sensors; 2019; 19: 2360. 10.3390/s19102360 PMC6566546 31121960

[36] ElMasry G, Sun DW, Allen P (2011). Non-destructive determination of water- holding capacity in fresh beef by using NIR hyperspectral imaging. Food Res Int.

[37] ElMasry G, Mandour N, Wagner M, Demilly D, Verdier J, Belin E, Rousseau D (2019). Utilization of computer vision and multispectral imaging techniques for classification of cowpea (*Vigna unguiculata*) seeds. Plant Methods.

[38] EIMasry G, Mandour N, Al-Rejaie S, Belin E, Rousseau D. Recent applications of multispectral imaging in seed phenotyping and quality monitoring-an overview. Sensors. 2019b; 19:1090. 10.3390/s19051090 PMC6427362 30836613

[39] ElMasry G, Nakazawa N, Okazaki E, Nakauchi S (2016). Non-invasive sensing of freshness indices of frozen fish and fillets using pretreated excitation- emission matrices. Sensors Actuators B Chem.

[40] Cortes C, Vapnik V (1995). Support vector network. Machine Learning.

[41] Devos O, Ruckebusch C, Durand A, Duponchel L, Huvenne JP (2009). Support vector machines (SVM) in near infrared (NIR) spectroscopy: focus on parameters optimization and model interpretation. Chemometrics Intelligent Laboratory Syst.

[42] Xie L, Ying Y, Ying T (2009). Classification of tomatoes with different genotypes by visible and short-wave near-infrared spectroscopy with least-squares support vector machines and other chemometrics. J Food Eng.

