# Supplementary figures and images for: Non-destructive identification of single hard seed via multispectral imaging analysis in six legume species
Source: Plant Methods. 2020 Aug 26;16:116. doi: 10.1186/s13007-020-00659-5 (PMC7448449; doi:10.1186/s13007-020-00659-5)

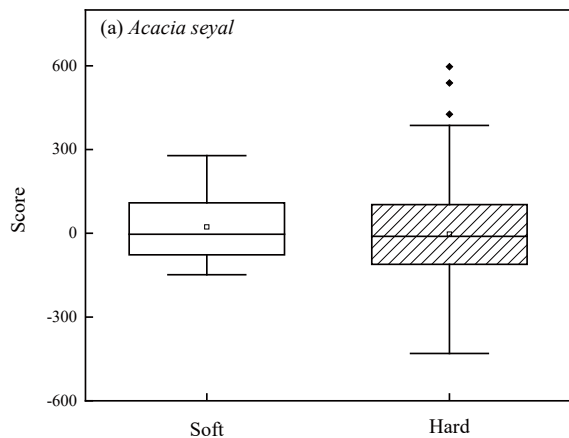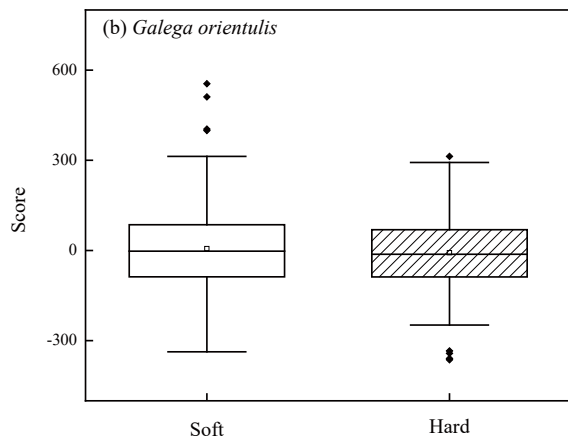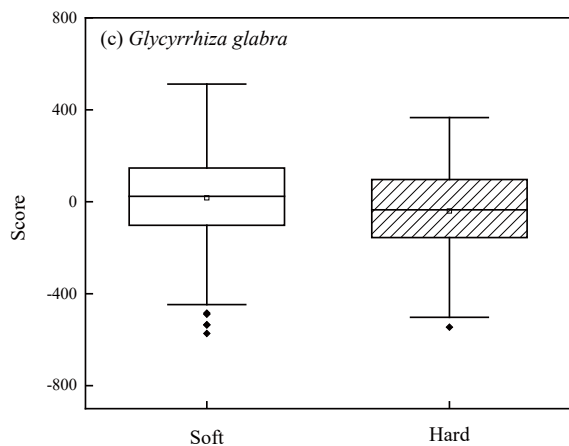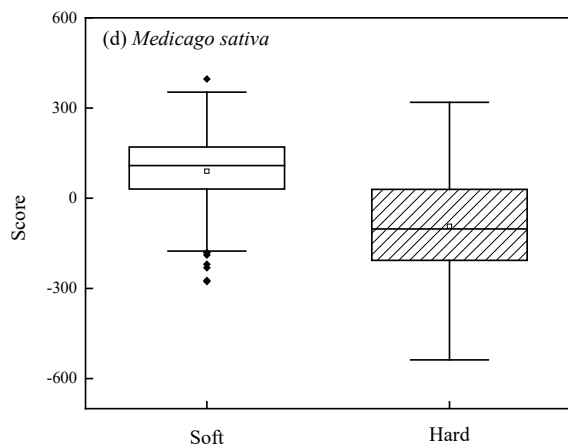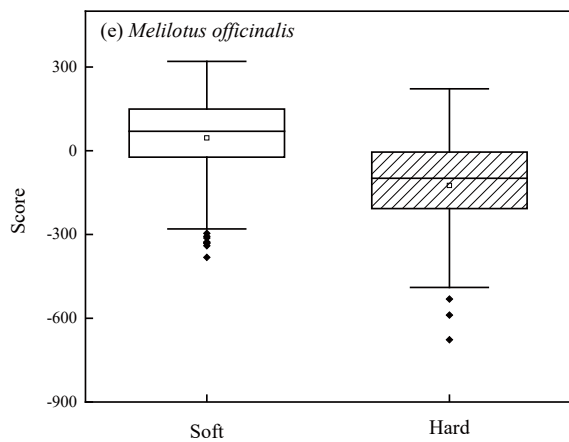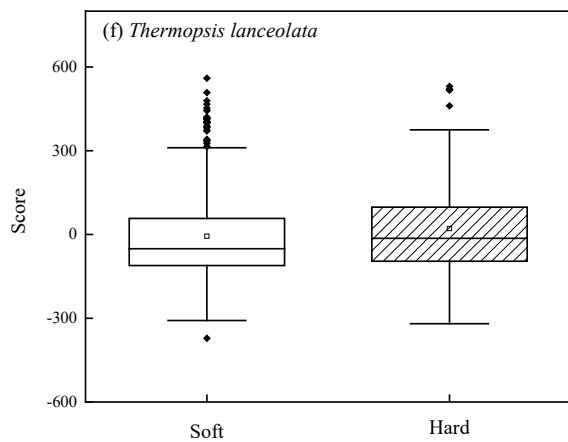

Supplement: Supplementary file 1 — Additional file 1: Figure S1. First two principal components score for hard and soft seeds. [file 13007_2020_659_MOESM1_ESM.pdf]

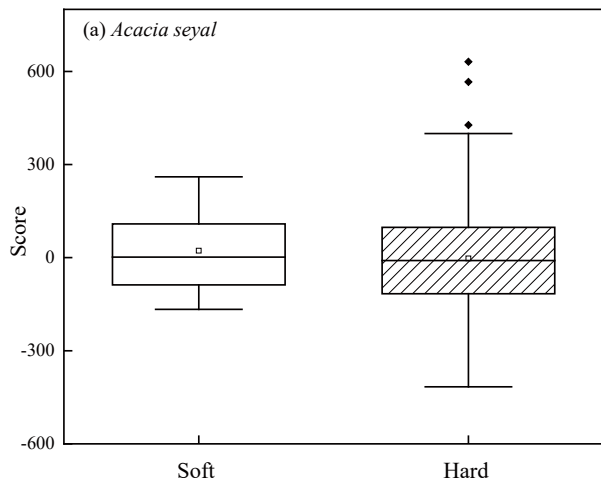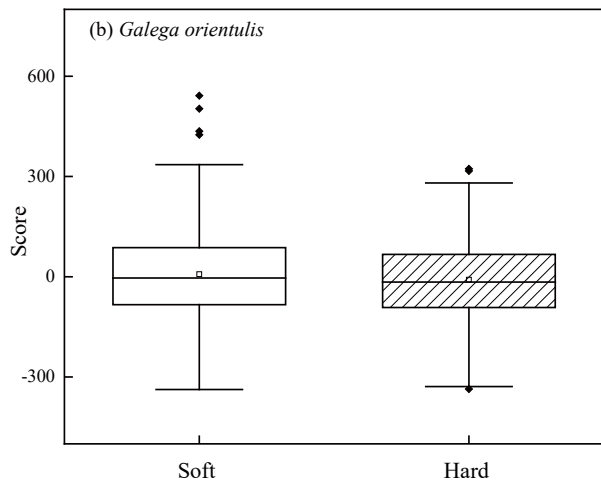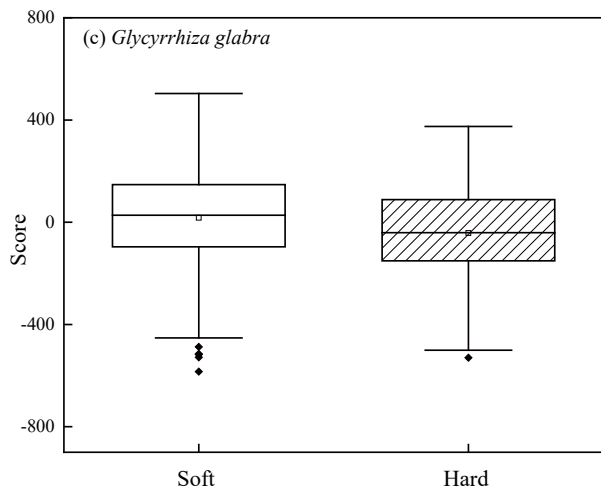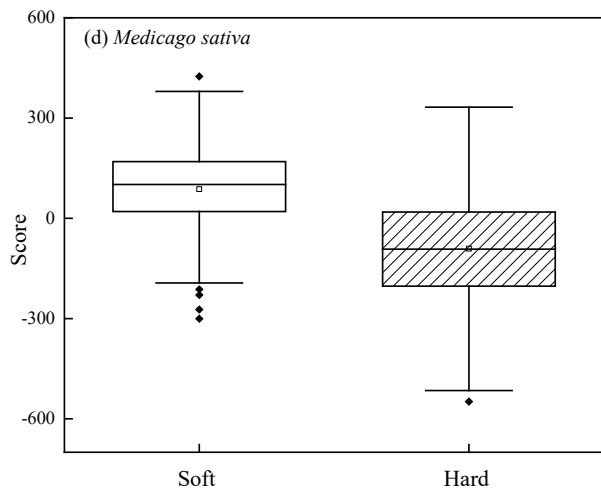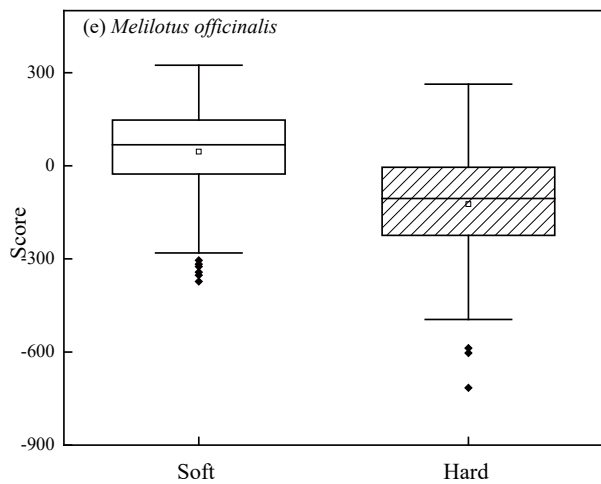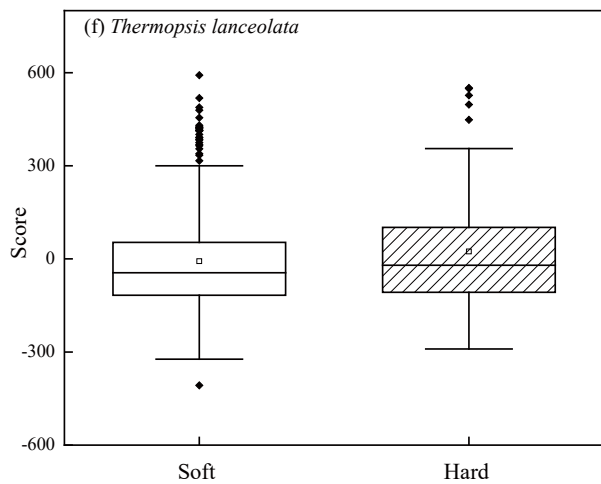

Supplement: Supplementary file 2 — Additional file 2: Figure S2. First three principal components score for hard and soft seeds. [file 13007_2020_659_MOESM2_ESM.pdf]

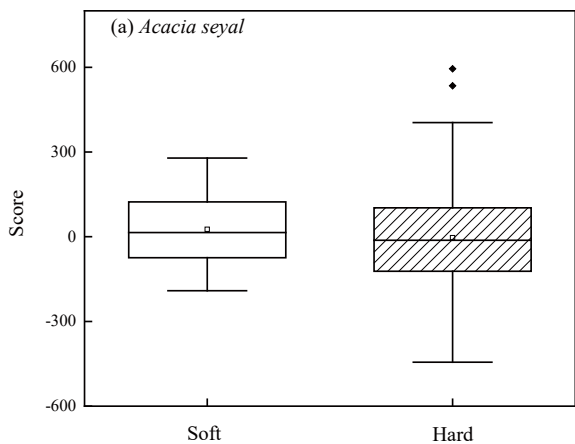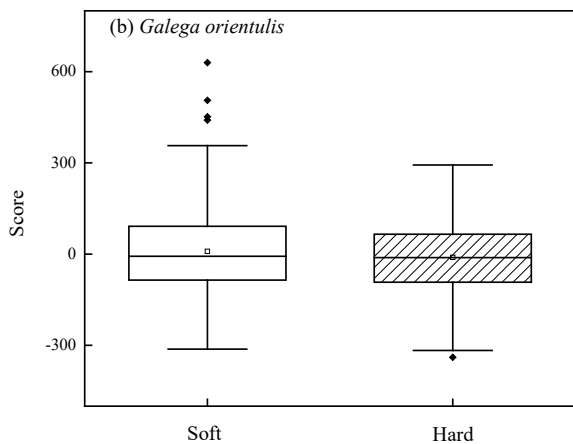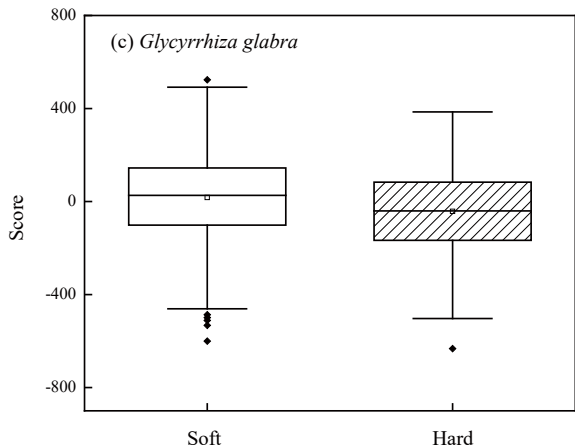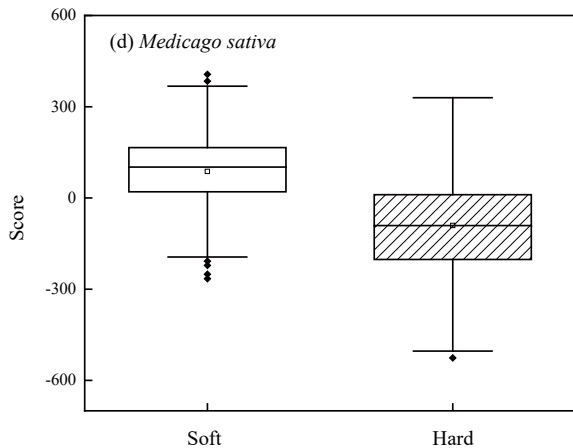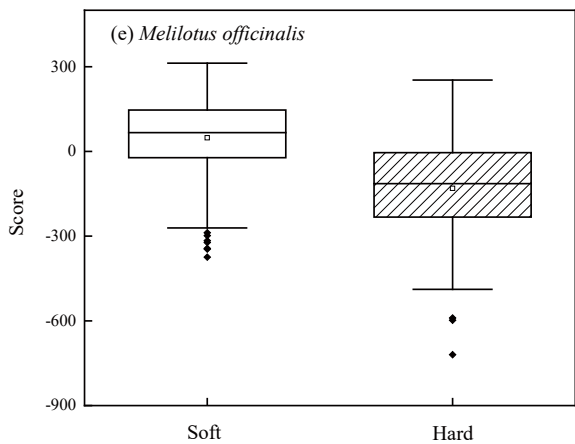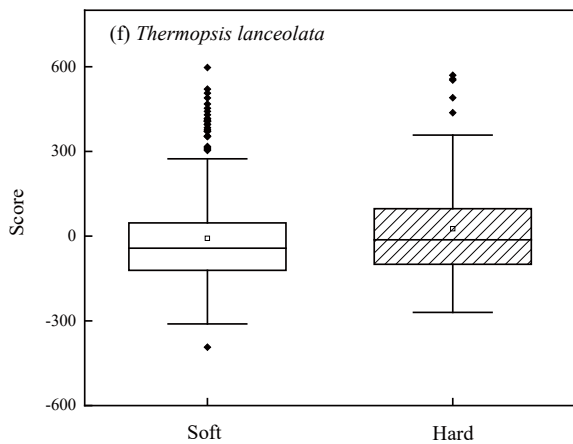

Supplement: Supplementary file 3 — Additional file 3: Figure S3. First ten principal components score for hard and soft seeds. [file 13007_2020_659_MOESM3_ESM.pdf]
