# Supplementary material for: Non-destructive identification of single hard seed via multispectral imaging analysis in six legume species
Source: Plant Methods. 2020 Aug 26;16:116. doi: 10.1186/s13007-020-00659-5 (PMC7448449; doi:10.1186/s13007-020-00659-5)
Supplement: Supplementary file 4 — Additional file 4: Table S1. List of the 15 extracted variables from multi spectral images. [file 13007_2020_659_MOESM4_ESM.docx]

Additional file 4: Table S1. List of the 15 extracted variables from multi spectral images.

| Name | Feature | Description |
| --- | --- | --- |
| Area(mm^2^) | Binary Feature | Area of blob |
| Length(mm) | Binary Feature | Length of blob bounding box. |
| Width(mm) | Binary Feature | Width of blob bounding box |
| Width/Length Ratio | Shape Feature | Ratio of width to length of the image oriented bounding box. |
| Compactness Circle | Shape Feature | Compactness of blob defined as 4*Area/(π*length^2), ratio of object area to the area of a circle with the same length (isolength quotient). |
| Compactness Ellipse | Shape Feature | Compactness of blob defined as 4*Area/(π*length*width), ratio of object area to the area of an ellipse with the same length and width (isolength quotient). |
| BetaShape a | Shape Feature | Parameter a of beta-ellipse fitted to blob mask. Parameter a corresponds to width of most pointed blob-end |
| BetaShape b | Shape Feature | Parameter b of beta-ellipse fitted to blob mask. Parameter b corresponds to width of least pointed blob-end |
| Vertical Skewness | Shape Feature | Skewness of blob mask around horizontal center-axis. |
| CIELab L* | Color Feature | Mean Luminance component of CIELab-color of blob |
| CIELab a* | Color Feature | Mean A-component of CIELab-color of blob |
| CIELab b* | Color Feature | Mean B-component of CIELab-color of blob |
| Saturation | Color Feature | Mean saturation of blob based on CIELab coordinates according to formulae: S = SQRT (A^2 + B^2) |
| Hue | Color Feature | Mean hue of blob based on CIELab coordinates according to formulae: H = ATAN (B/A) |
| Vertical Orientation | Texture Feature | Index of feature perpendicular anisotropy |
